# Polarization-insensitive optical coherence tomography using pseudo-depolarized reference light for mitigating birefringence-related image artifacts

**DOI:** 10.1117/1.JBO.29.11.116001

**Published:** 2024-11-04

**Authors:** Maria Varaka, Conrad W. Merkle, Lucas May, Sybren Worm, Marco Augustin, Félix Fanjul-Vélez, Hsiang-Chieh Lee, Adelheid Wöhrer, Martin Glösmann, Bernhard Baumann

**Affiliations:** a Medical University of Vienna, Center for Medical Physics and Biomedical Engineering, Vienna, Austria; b University of Cantabria, Biomedical Engineering Group, TEISA Department, Santander, Spain; c National Taiwan University, Graduate Institute of Photonics and Optoelectronics, Taipei, Taiwan; d National Taiwan University, Department of Electrical Engineering, Taipei, Taiwan; e Medical University of Vienna, Division of Neuropathology and Neurochemistry, Department of Neurology, Vienna, Austria; f Medical University of Innsbruck, Department of Pathology, Neuropathology and Molecular Pathology, Innsbruck, Austria; g University of Veterinary Medicine Vienna, VetCore Facility for Research, Imaging Unit, BioImaging Austria/CMI, Vienna, Austria

**Keywords:** optical coherence tomography, polarization, polarization artifacts, birefringence

## Abstract

**Significance:**

Optical coherence tomography (OCT) images are prone to image artifacts due to the birefringence of the sample or the optical system when a polarized light source is used for imaging. These artifacts can lead to degraded image quality and diagnostic information.

**Aim:**

We aim to mitigate these birefringence-related artifacts in OCT images by adding a depolarizer module in the reference arm of the interferometer.

**Approach:**

We investigated different configurations of liquid crystal patterned retarders as pseudo-depolarizers in the reference arm of OCT setups. We identified the most effective depolarization module layout for polarization artifact suppression for a spectral-domain OCT system based on a Michelson and a Mach–Zehnder interferometer.

**Results:**

The performance of our approach was demonstrated in an achromatic quarter-wave plate allowing the selection of a variety of sample polarization states. A substantial improvement of the OCT signal magnitude was observed after placing the optimal depolarizer configuration, reducing the cross-polarization artifact from 5.7 to 1.8 dB and from 8.0 to 1.0 dB below the co-polarized signal for the fiber-based Michelson and Mach–Zehnder setup, respectively. An imaging experiment in the birefringent scleral tissue of a post-mortem alpine marmot eye and a mouse tail specimen further showcased a significant improvement in the detected signal intensity and an enhanced OCT image quality followed by a drastic elimination of the birefringence-related artifacts.

**Conclusions:**

Our study presents a simple yet cost-effective technique to mitigate birefringence-related artifacts in OCT imaging. This method can be readily implemented in existing OCT technology and improve the effectiveness of various OCT imaging applications in biomedicine.

## 1 Introduction

Optical coherence tomography (OCT) is a non-invasive imaging modality capable of obtaining real-time cross-sectional images of the tissue with micrometer resolution.^1^ OCT imaging has become an important tool in medical diagnostics and found applications in various medical fields such as dermatology for skin cancer diagnosis,^2^^,^^3^ cardiology and gastroenterology for the diagnosis of plaques and esophagus malignancies,^4^^–^^8^ and dentistry for monitoring demineralization or remineralization processes in tooth lesions.^9^^–^^11^ Ophthalmology is the medical domain where OCT is used as a standard diagnostic method in everyday clinical routine. It stands as a robust diagnostic tool for common eye diseases such as glaucoma, age-related macular degeneration, or diabetic retinopathy.^12^^,^^13^

Despite its wide range of applications in biomedicine and its high-resolution imaging capabilities, OCT also has limitations. Modern OCT techniques often use light sources which emit highly polarized light.^14^^–^^16^ As OCT is based on interfering sample light with a reference beam, only the component of the light backscattered from the sample which is co-polarized with the polarization state of the reference beam contributes to the interference signal. Conversely, cross-polarized sample light cannot interfere with the reference beam, thus leading to OCT signal degradation and even complete signal loss. This signal loss is captured in the image as image artifacts such as stripes or signal fading.^17^^,^^18^ One cause of these birefringence artifacts is inherently birefringent tissues that alter the polarization state of light and thereby may reduce interference fringe visibility. Birefringence is caused for instance by collagen fibers forming structures in tissues such as muscles, skin, tendons, or sclera,^19^^–^^23^ whereas nerve tissue structures such as white brain matter or the retinal nerve fiber layer exhibit birefringence owing to the microstructure of the nerve fibers.^24^^–^^26^ Studies focused on ocular imaging revealed birefringence-related artifacts in the sclera which mimicked scleral vessels or low-intensity bands.^27^^–^^29^ Modified or damaged tissue morphology can also affect its birefringence and thus significantly change the polarization state of backscattered light.^30^^,^^31^ Another cause of artifacts, in addition to the aforementioned structural factors related to tissue, is the birefringence of the optical components in an OCT system. Especially in fiber-based optical systems and endoscopic systems, bending or twisting of the fiber components may drastically change the polarization state of propagating light. For instance, OCT images and statistical analysis from endoscopic tendon imaging revealed a strong correlation between bending of the catheter and birefringence alteration.^32^

Different approaches based on polarization-sensitive OCT (PS-OCT) have been presented for removing these birefringence-related artifacts.^27^^,^^29^^,^^33^ PS-OCT can differentiate among various polarization states, providing detailed information about tissue birefringence. As it detects complete Jones vectors, Stokes vectors, Jones matrices, or Müller matrices, PS-OCT can provide reflectivity imaging devoid of polarization artifacts, in addition to image data of other polarization quantities. However, PS-OCT requires rather complex system layouts and sophisticated data processing.^34^^,^^35^ Polarization diversity detection in Fourier-domain OCT systems has also demonstrated the ability to mitigate polarization artifacts related to sample or fiber birefringence.^36^ Although polarization diversity OCT systems are typically much less technologically demanding than their PS-OCT counterparts, they usually require at least one additional detection channel for orthogonally polarized light. In this paper, we propose a simple yet cost-effective method to eliminate the OCT image artifacts, which improves on our recently presented polarization-insensitive OCT (PinS-OCT) approach.^37^ By placing a depolarization module based on patterned retarders in the reference arm of an OCT system using a polarized light source, the severity of birefringence artifacts was greatly reduced. We implemented our PinS-OCT method in Mach–Zehnder and Michelson interferometer layouts and demonstrated its performance both for a technical sample, which enabled testing of a variety of sample polarization states, and in birefringent biological tissue.

## 2 Materials and Methods

### 2.1 Evaluation of Depolarization Modules

The key component of our PinS-OCT approach is a patterned retarder, which acts as a pseudo-depolarizer. In our previous research, the depolarizing element was placed in the source arm of a fiber-based Michelson interferometer and effectively used to eliminate the OCT image artifacts.^37^ Here, we propose an alternative method where the depolarizer module was placed in the reference arm of a fiber-based Michelson interferometer and a fiber-based Mach–Zehnder interferometer. The rationale for choosing the reference arm for the implementation presented here lies in the fact that this approach offers additional advantages over the previously published source arm approach, particularly for fiberized OCT interferometers. In fiber-based OCT systems, the implementation of the depolarizer configuration in the reference arm does not need an additional free-space segment as was the case in our previous fiber-based source arm approach. Therefore, the proposed method here not only allows for a more straightforward implementation of the depolarization stage but also comes with less power loss in the source arm of the system. Necessitating to couple out of and back into a single-mode fiber, the free-space source arm approach presented in Ref. 37 led to an additional ∼50% loss of the input power. This power is not sacrificed in free-space optical systems nor in the reference arm–based approach demonstrated here. In addition to the previously used liquid crystal polymer depolarizer (DPP25-B, Thorlabs, Newton, New Jersey, United States; henceforth called depolarizer DP1), we also investigated a custom-made liquid crystal depolarizer (PLCC-02579, Thorlabs; henceforth called depolarizer DP2) similar to DP1 but specified to provide half the retardation of the DP1. In brief, the liquid crystal polymer depolarizers DP1 and DP2 are patterned microretarders that modify the polarization of input light to contain spatially varying polarization states over the beam cross-section.

**Fig. 1:**
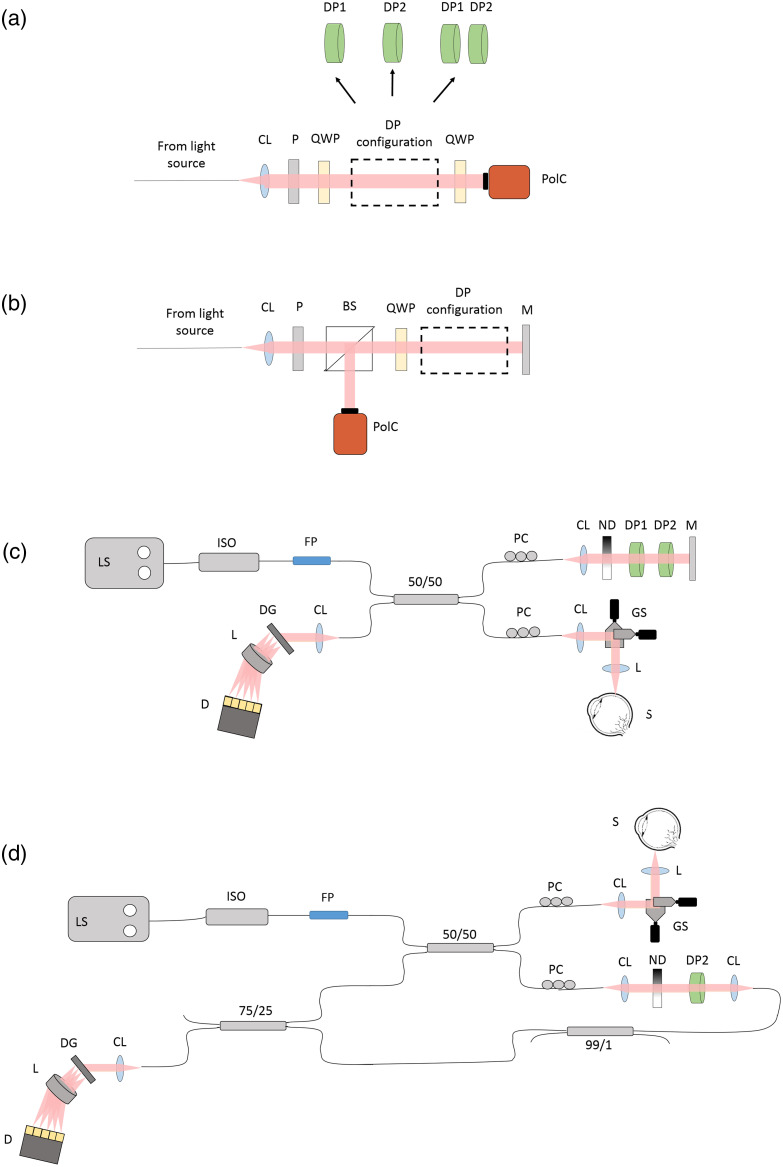
Schematic representation of the experimental setups. Experimental setup for the measurement of the polarization effects of different combinations of the two patterned retarders for (a) single pass and (b) double pass. (c) Schematic representation of the fiber-based Michelson interferometer. (d) Schematic representation of the fiber-based Mach–Zehnder interferometer. CL, collimator lens; P, polarizer; BS, beam splitter; QWP, achromatic quarter wave plate; DP1 and DP2, liquid crystal polymer depolarizers; M, mirror; PolC, polarization camera; LS, light source; ISO, isolator; FP, fiber polarizer; 50/50, 99/1, and 75/25, single-mode fiber couplers; PC, polarization controller; L, scan lens; ND, neutral density filter; GS, X−Y galvanometer scanners; S, sample; DG, diffraction grating; D, line-scan camera.

Different configurations of the two depolarizers were tested. To evaluate the overall depolarization effect caused by each pseudo-depolarizer configuration, two test setups with a polarization-sensitive camera as a detector were implemented [Figs. 1(a) and 1(b)] similar to our previously presented polarization microscopy approach.^38^ These setups were used to test the depolarizer configuration after single pass [Fig. 1(a)] and double pass [Fig. 1(b)] through the microretarders. The light source used in this free-space test setup featured the same superluminescent diode as in our PinS-OCT configurations detailed in Secs. 2.2 and 2.3. We placed a Glan–Thompson polarizer after the collimator lens to make the input light beam vertically polarized. After passing a non-polarizing beam splitter cube, an achromatic quarter wave plate (QWP) oriented at 45 deg converted the beam into a circularly polarized state. One or both depolarizers were placed as the device under test between the QWP and the mirror of the test setup. The beam reflected by the mirror passed through the depolarizers and the QWP again, and the signal reflected by the beam splitter was detected by a polarization-sensitive monochrome camera (Thorlabs, CS505MUP1). This camera features a grid polarizer where each pixel is covered with one of four different polarizers with orientations of 0, 90, 45, and −45  deg. The Jones vector at the camera can be described by

$$J=\frac{1}{2}M_{\mathrm{QWP}}(45\;\deg)M_{S}(\delta ,\theta {)}^{2}M_{\mathrm{QWP}}(45\;\deg)[\begin{array}{c} 0\\ 1 \end{array}],$$ (1)

where $M_{\mathrm{QWP}}(45\;\deg)$ denotes the Jones matrix of the QWP oriented at 45 deg, and $M_{S}(\delta ,\theta )$ is the Jones matrix of a general linear retarder with retardation δ and fast axis orientation θ. By calculating Eq. (1) similar to Ref. 39, the output Jones vector becomes

$$J=\frac{\sqrt{R}}{2}[\begin{array}{c} \cos\;\delta e^{-i\delta }\\ \sin\;\delta e^{i(\pi -\delta -2\theta )} \end{array}],$$ (2)

where R is the sample reflectivity. Sampled by the four different polarizer orientations, namely, at 0, 90, 45, and −45  deg, the intensities at the respective polarizers are expressed by

$$I_{0\;\deg}=\frac{R}{4}\;\cos^{2}\;\delta ,$$ (3)

$$I_{90\;\deg}=\frac{R}{4}\;\sin^{2}\;\delta ,$$ (4)

$$I_{45\;\deg}=\frac{R}{8}-\frac{R}{4}\;\sin\;\delta \;\cos\;\delta \;\cos\;2\theta ,$$ (5)

$$I_{-45\;\deg}=\frac{R}{8}+\frac{R}{4}\;\sin\;\delta \;\cos\;\delta \;\cos\;2\theta .$$ (6)

From these four intensities, the sample reflectivity R, (single pass) phase retardation δ, and fast axis orientation θ can be computed as

$$R=4(I_{0\;\deg}+I_{90\;\deg})=2(I_{0\;\deg}+I_{90\;\deg}+I_{45\;\deg}+I_{-45\;\deg}),$$ (7)

$$\delta =\arctan(\frac{\sqrt{I_{90\;\deg}}}{\sqrt{I_{0\;\deg}}}),$$ (8)

$$\theta =\frac{1}{2}\;\arccos(\frac{I_{-45\;\deg}-I_{45\;\deg}}{2\sqrt{I_{0\;\deg}}\sqrt{I_{90\;\deg}}}).$$ (9)

The above equations were used for the double-pass configuration [Fig. 1(b)]. For the single-pass configuration shown in Fig. 1(a), the factor 1/2 in Eq. (1) was not taken into account because, here, the light does not pass through the beam splitter, which reduces the intensity of the reference beam by a factor of 2. Furthermore, by simply substituting $M_{S}$ in Eq. (1) with a sample Jones matrix having half the retardation of the double-pass case, $\delta^{\prime }=\delta /2$, Eq. (1) and the following equations can be employed for the single-pass layout, again assuming a linear retarder. Consequently, the retardation for the single-pass configuration can be computed as half of the double-pass retardation

$$\delta^{\prime }=\frac{1}{2}\;\arctan(\frac{\sqrt{I_{90\;\deg}}}{\sqrt{I_{0\;\deg}}}).$$ (10)

Using δ and θ, the Jones matrix of the linear retarder $M_{\mathrm{DP}}(x,y)$, describing the birefringence effect for every quadruplet of pixels, can be calculated. By computationally propagating a vertical polarization state $[\begin{array}{cc} 0 & 1 \end{array}{]}^{T}$ through $M_{\mathrm{DP}}(x,y)$ and $M_{\mathrm{DP}}(x,y{)}^{2}$ for single- and double-pass configurations, respectively, the set of Jones vector after the depolarizer DP was calculated. After transforming the Jones vectors at every spatial position to their corresponding sets of Stokes parameters $[S_{0},S_{1},S_{2},S_{3}{]}^{T}$, the degree of polarization was computed as

$$\mathrm{DOP}=\frac{\sqrt{{\overset{¯}{S_{1}}}^{2}+{\overset{¯}{S_{2}}}^{2}+{\overset{¯}{S_{3}}}^{2}}}{\overset{¯}{S_{0}}},$$ (11)

where overbars denote spatial averaging across the beam cross-section. The degree of polarization (DOP) was calculated similarly to the degree of polarization uniformity (DOPU) in PS-OCT,^40^ albeit across the whole beam cross-section instead of within a small spatial kernel.

### 2.2 Michelson Interferometer

A spectral domain OCT setup based on a fiber-based Michelson interferometer was modified to demonstrate the polarization-insensitive OCT imaging approach [Fig. 1(b)]. A superluminescent diode (Superlum, Cork, Ireland, SLD-371-HP2) with a central wavelength of 840 nm and a full width at half maximum of 50 nm was used as a light source. An isolator was used to protect the light source from harmful back reflections. A fiber polarizer (OZ Optics, Ottawa, Canada) was connected after the isolator to polarize the light exiting the light source. A 2×2 wideband 50/50 single-mode fiber coupler (Thorlabs, TW850R5A2) was used to split the light beam into the reference and sample arms. The sample arm contains an X−Y galvanometer scanner (Thorlabs, GVS002) and an achromatic scan lens (f=30  mm), whereas in the reference arm, a collimator with 7.5-mm focal length and a neutral density (ND) filter were placed. The reference beam diameter was 1.8 mm, and the period of the retardation pattern produced by the pseudo-depolarizers measured using the polarization-sensitive camera amounted to 1.5 mm. Consequently, the reference beam covered slightly more than one cycle of the retardation pattern. According to the DOP calculations and measurements in a technical sample in Secs. 2.1 and 2.4, respectively, a combination of both depolarizers was found to be the most effective configuration for image artifact reduction. The polarization controllers in the reference and sample arms were initially aligned without the depolarization module in the reference arm and in such a way that the maximum interference fringe visibility was achieved. The depolarizers were placed in the reference arm between the ND filter and the mirror, with the patterns oriented at 60 deg with respect to each other. This orientation of the depolarizer patterns was empirically found to yield the most favorable artifact suppression (data not shown). First, the depolarizer DP1 was introduced into the reference arm and was rotated until the highest interference signal amplitude was achieved. Then, the second depolarizer DP2 was placed behind DP1 and was again rotated until the highest signal was obtained. The final orientations of depolarizers DP1 and DP2 were at 45 and 105 deg, respectively, and remained fixed for all the setups shown in Fig. 1. The output of the single-mode fiber coupler was connected to the spectrometer. The spectrometer comprised a 1800-line/mm transmission grating (Wasatch Photonics, Logan, Utah, United States), a 100-mm focal length f-theta lens (Cloudray, Nanjing, China), and a line-scan camera (Vieworks) with 4096 pixels operated at a rate of 50 kHz. The system offered a depth range of 5.72 mm (in air); an axial resolution of 7.6  μm (in air); a sensitivity of 93.3 and 95.1 dB with a sample arm power of 860  μW with and without the pseudo-depolarizers in the reference arm, respectively; and a signal-to-noise ratio (SNR) roll-off of 6.5 dB over a distance of 3 mm (in air).

### 2.3 Mach–Zehnder Interferometer

A fiber-based Mach–Zehnder interferometer setup with a spectral domain detection [Fig. 1(c)] was built to demonstrate the effect of depolarizers on image artifact reduction for an optical layout frequently used for swept-source OCT. Unlike the Michelson interferometer, the light passed only once through the depolarizer in the reference arm. For a single pass and according to the results described in Secs. 3.1 and 3.2, the most effective reduction of birefringence artifacts could be achieved by placing only the pseudo-depolarizer DP2 in the setup. The polarization controllers were aligned in the same way as in the previous setup, whereas in addition to the 50/50 fiber coupler, also a 99/1 and a 75/25 single-mode fiber coupler were connected to the system. The 1% tap of the 99/1 fiber coupler was used for the reference arm beam alignment and the 25% of the 75/25 coupler for the system power monitoring. A pair of identical collimators (f=7.5  mm) was placed in the reference arm of the interferometer. The light source as well as the spectrometer characteristics remained the same as mentioned in Sec. 2.2. A sensitivity of 92.7 and 93.3 dB were measured with and without the depolarization stage in the reference arm of the system, respectively.

### 2.4 Polarization-Insensitive OCT System Validation and Sample Handling

To validate the systems’ robustness with the depolarizers in the reference arm, we placed an achromatic quarter-wave plate and a mirror in the sample position. In addition, a variable ND filter was inserted into the sample arm to avoid saturation of the spectrometer camera. The measured sample arm power after the ND filter implementation was 15  μW for both Michelson and Mach–Zehnder interferometers. The QWP was mounted in a rotation mount such that the sample polarization could be rotated by twice the QWP’s angle of orientation upon double-passing the QWP. By rotating the QWP in the range of 0 to 180 deg with a step size of 15 deg, we acquired axial scans for every QWP position. By measuring the signal intensity at the peak of the coherence function, this enabled an investigation of the signal intensity from different sample polarization states interfering with the reference beam. We followed the same procedure with and without the depolarization module in the reference arm. For the arrangement combining the two pseudo-depolarizers in the Michelson interferometer layout, additional measurements were conducted to test if the depolarization performance was affected by the relative orientation between the two depolarizers as well as by their order (first DP1 then DP2 and vice versa). Using a 45-deg relative orientation as the starting point similar to Sec. 2.2, we were rotating the two depolarizers in the range of 0 to 90 deg with a step size of 15 deg. For testing the configuration DP1–DP2, DP2 was rotated first, whereas DP1 was rotated first for the inverse order, DP2–DP1. For each depolarizer angle combination, the signal amplitude was measured for QWP positions in 15-deg steps between 0 and 180 deg.

To demonstrate PinS-OCT imaging in biological tissue, a formalin-fixed alpine marmot eye specimen obtained from the University of Veterinary Medicine, Vienna, and a formalin-fixed *ex vivo* mouse tail sample were investigated. Image acquisition was conducted sequentially with and without the depolarization module in both the Michelson and the Mach–Zehnder interferometer–based OCT layout.

## 3 Results

### 3.1 Influence of Sample Polarization State on the Signal Intensity and Depolarization Stage Selection

**Fig. 2:**
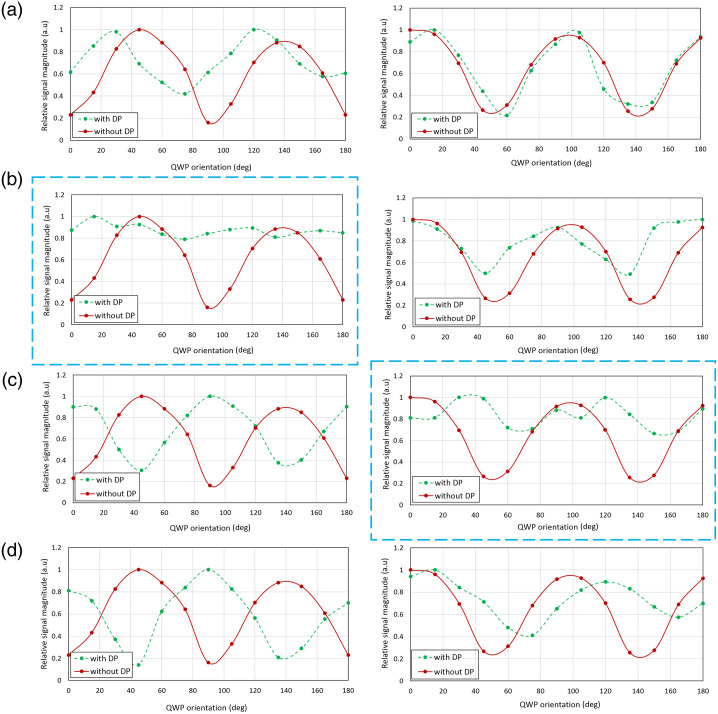
Interference signal dependence on QWP orientation with and without the depolarization stage featuring (a) DP1, (b) DP2, (c) DP1-DP2, and (d) DP2-DP1 in the reference arm of the Mach–Zehnder (left column) and Michelson interferometer (right column). The dashed blue boxes indicate the selected pseudo-depolarizer configurations for both setups.

We first investigated the robustness of our PinS-OCT approach to different sample polarization states dialed in by rotating a QWP in front of a mirror in the sample position. Figure 2 shows the dependence of the interference signal intensity on the QWP orientation for the four depolarization stage configurations in the reference arm and for both Mach–Zehnder and Michelson interferometer layouts. A strong dependence of the interference signal on the different QWP orientations was observed for the standard OCT configuration (i.e., without a depolarization module in the reference arm). Conversely, the interference signal remained more stable for the QWP orientations when the selected depolarizer configurations were employed in the reference arm of both interferometers. By calculating the relative signal fluctuations as $10\;\log(I_{\max}/I_{\min})$, a notable improvement was observed after placing the optimal depolarizer configuration raising the cross-polarized signal from 5.7 to 1.8 dB below the maximum (co-polarized) signal and from 8.0 to 1.0 dB for the Michelson and Mach–Zehnder setup, respectively. Additional measurements were conducted to explore the dependence on the relative orientation and the order between the two pseudo-depolarizers. For the configuration DP1–DP2, the performance was found to be sensitive to the orientation among the depolarizers. The relative signal fluctuations ranged between 3.1 and 1.8 dB for 45- and 60-deg (selected configuration) relative orientation, whereas for the inverse order DP2–DP1, by rotating the pseudo-depolarizer DP1, this range increased to 7.1 and 3.2 dB for 30- and 60-deg relative orientation, respectively. Using the decrease in the relative signal fluctuations as a metric, the application of one depolarizer (namely, DP2) appeared best suited for single-pass application in the Mach–Zehnder interferometer where the light passes only once through the depolarizer, whereas the configuration DP1–DP2 with a relative orientation of 60 deg was selected for the implementation using a Michelson interferometer layout, which requires double pass through the depolarization stage.

### 3.2 Depolarization Effect of the Pseudo-Depolarizer Configurations

**Fig. 3:**
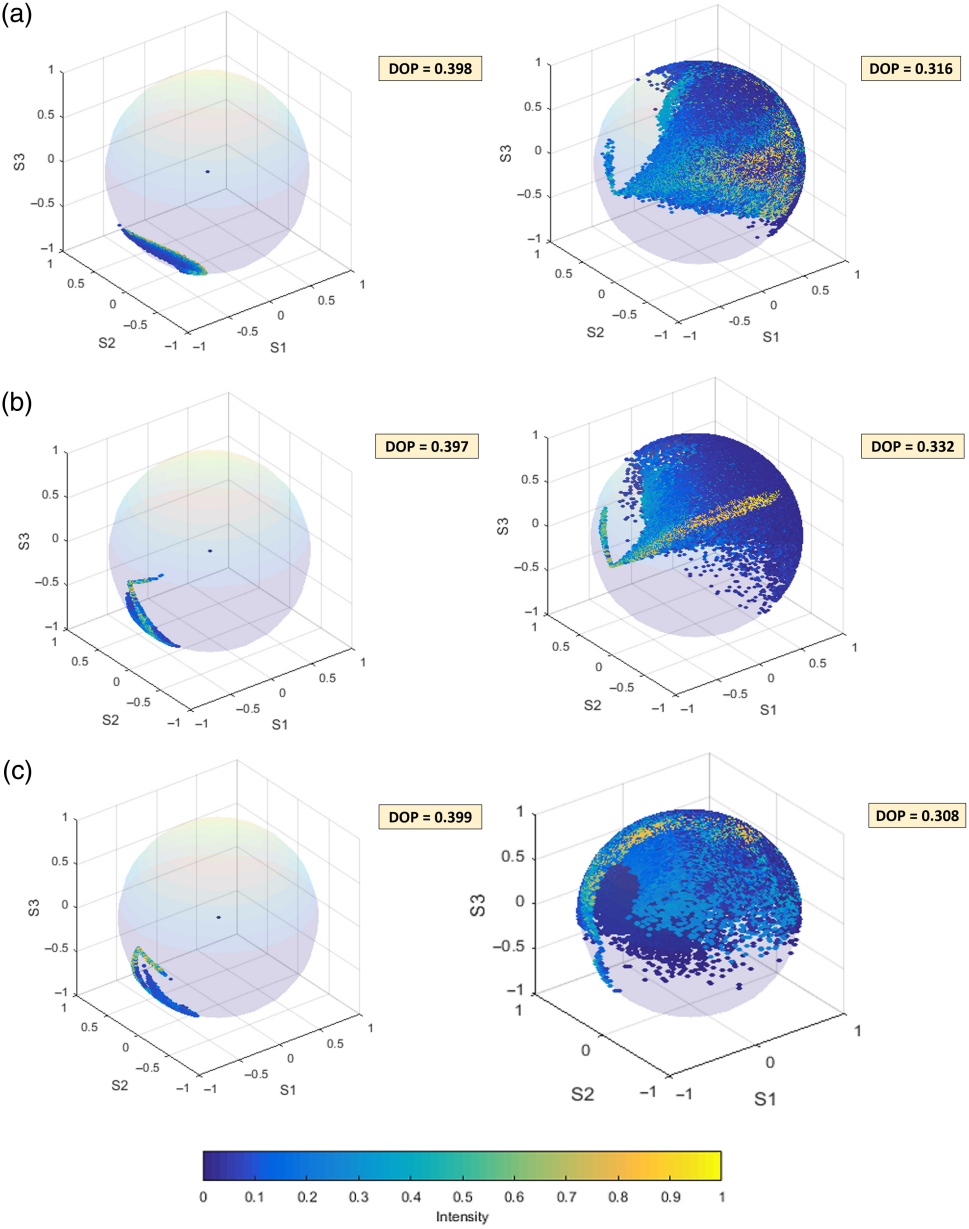
Schematic representation of the polarization state distributions on the Poincaré sphere for single pass (left column) and double pass (right column) of (a) DP1, (b) DP2, and (c) both DP1 and DP2. The calculated DOP values are noted in the top right corner of each case. The color bar on the bottom represents the normalized intensity.

Figures 3(a)–3(c) show a Poincaré sphere representation of the polarization state distribution across the beam cross-section after passing the depolarization module once (left column) or twice (right column). For the measurements investigating the single-pass performance of the depolarizers, we used the setup depicted in Fig. 1(a) where we placed DP1 alone [Fig. 2(a)], DP2 alone [Fig. 2(b)], and DP1 and DP2 together [Fig. 2(c)], with the DP2 placed behind the DP1, between two QWPs. For evaluating the double-pass performance, we used the setup of Fig. 1(b), and the aforementioned depolarizer configurations were implemented between the QWP and the mirror. The polarization state distributions for the three different depolarizer configurations are displayed on the Poincaré sphere as normalized Stokes vectors $[S_{1}/S_{0},S_{2}/S_{0},S_{3}/S_{0}{]}^{T}$ for every pixel along with the corresponding DOP which was estimated across the beam cross-section. Note that the vertical input state corresponding to −1 on the S1 axis is rendered into a large continuum of polarization states across the output beam. Also note how higher-intensity beam components (data points mapped in bright colors) describe a rather confined trace on the Poincaré sphere, whereas lower-intensity components (bluish data points) are rather widely spread across the sphere. Each specific depolarizer configuration leads to different DOP values for both setups. Interestingly, the DOPs for double pass are lower compared with single pass, with the biggest difference being noticed in the third case, and although DOP was very similar for all single-pass configurations, a marked DOP difference among the double-pass measurements was observed.

### 3.3 Imaging Scleral Tissue and Mouse Tail with Polarization-Insensitive OCT

**Fig. 4:**
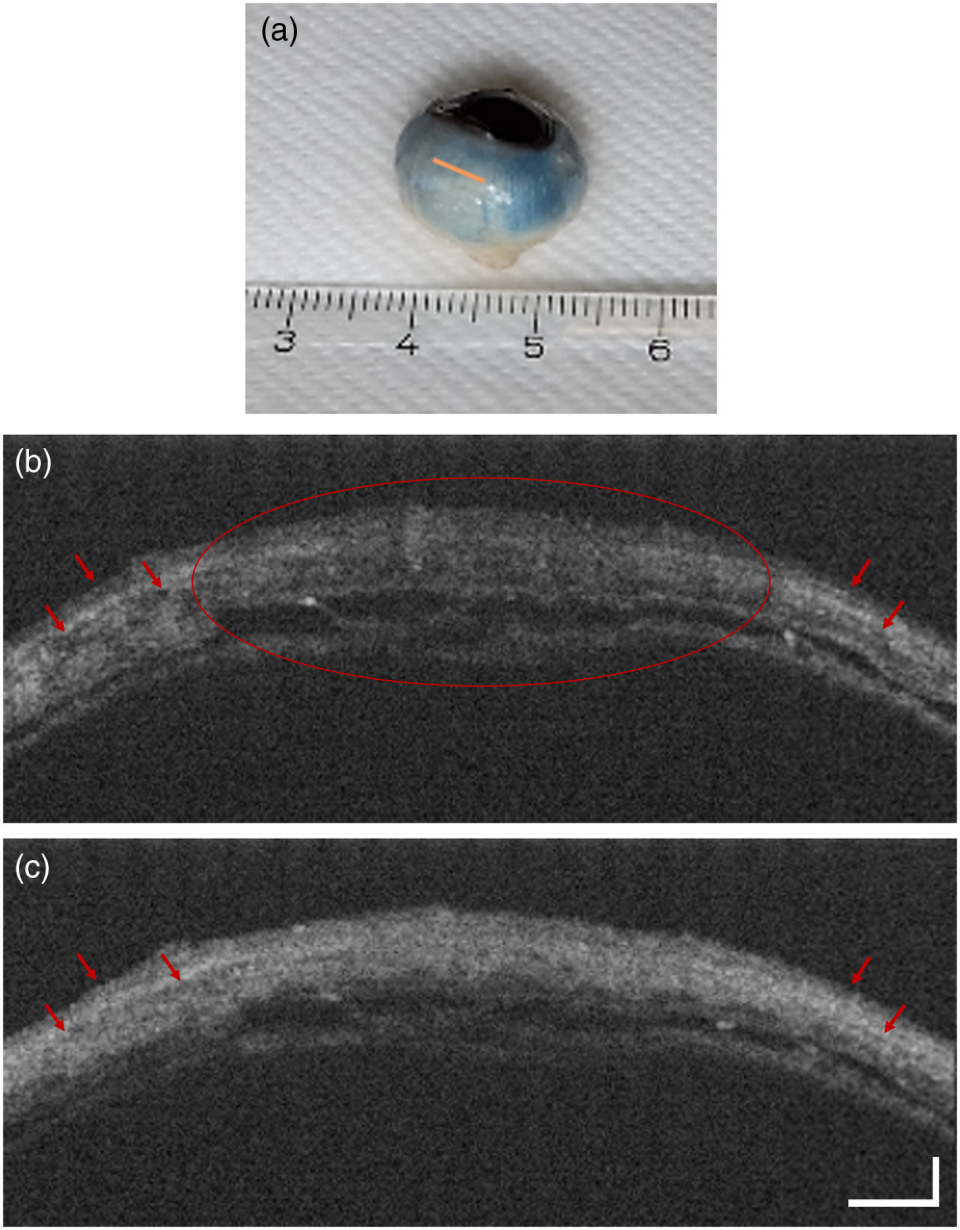
OCT imaging of the sclera of an alpine marmot specimen with polarization-insensitive OCT implemented in a fiber-based Michelson interferometer. Panel (a) shows the scanned region of the scleral tissue (orange line). (b) and (c) OCT images of the scleral tissue without and with the depolarization module in the reference arm, respectively. Birefringence-related artifacts (red arrows) are drastically reduced, and the signal in the central area of the tissue (red circle) is strongly improved. The scale bars in panel (c) apply for both figures. The horizontal bar represents 750  μm, and the vertical bar represents 350  μm.

**Fig. 5:**
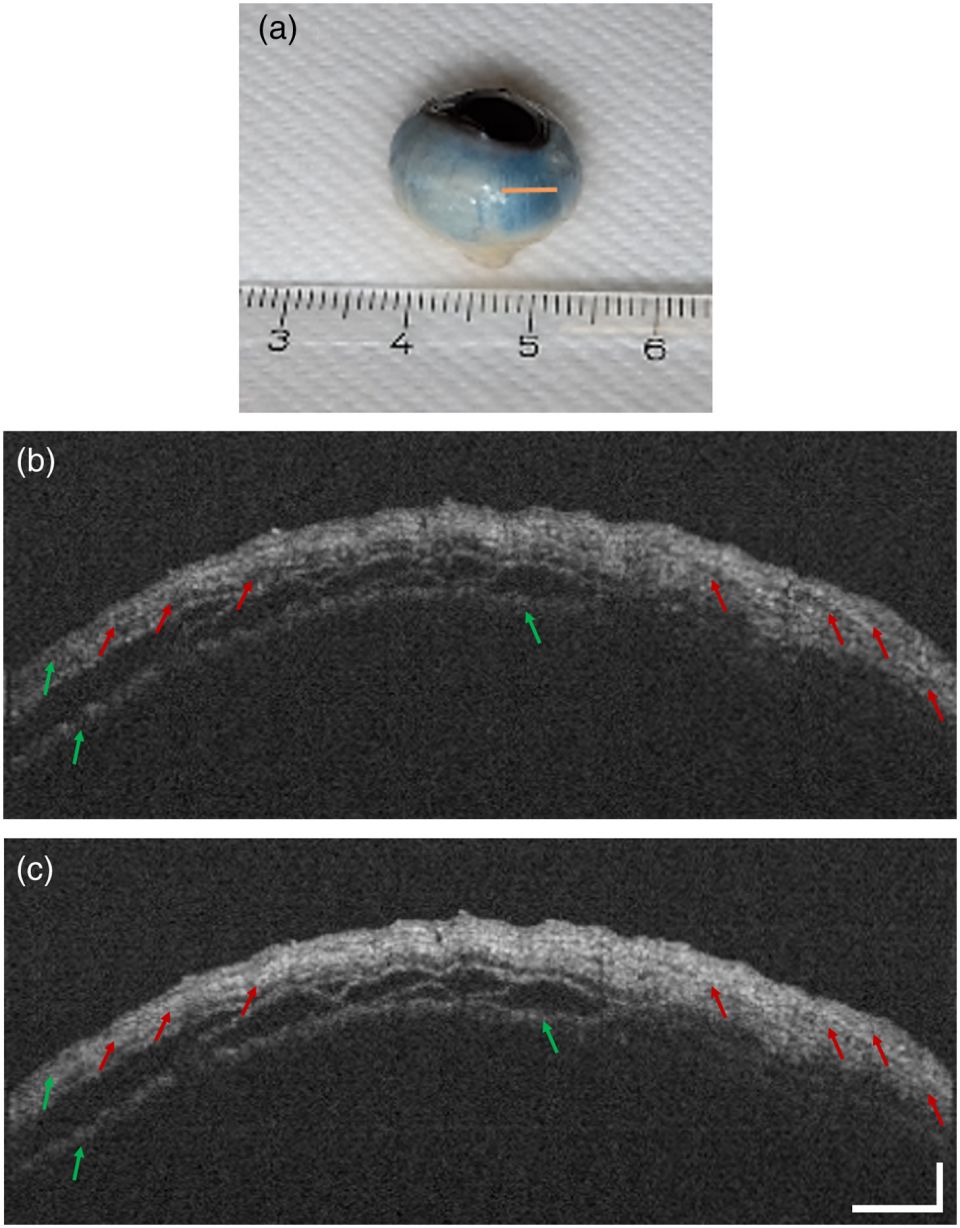
OCT imaging of the alpine marmot sclera specimen with polarization-insensitive OCT implemented in a fiber-based Mach–Zehnder interferometer. The orange line in panel (a) indicates the scanning region in the scleral tissue. (b) and (c) OCT B-scans of the scleral tissue without and with the depolarization module in the reference arm, respectively. The hypointense artifacts (red arrows) are diminished, whereas the tissue structure appears clearer after the depolarizer is implemented. Faded layers of the tissue (green arrows) seem more distinct. The scale bars in panel (c) apply for both figures. The horizontal bar represents 750  μm, and the vertical bar represents 350  μm.

Finally, we demonstrate PinS-OCT for imaging birefringent tissue. First, OCT images of the post-mortem alpine marmot eye were acquired with the standard Michelson interferometer configuration. Figures 4(a) and 5(a) show the region close to the corneoscleral limbus selected for imaging. When the depolarization module was absent, the scleral tissue exhibited characteristic hypointense artifacts due to the birefringence induced by collagenous structures [Fig. 4(b)]. Conversely, OCT imaging using the depolarization module in place mitigated these artifacts, resulting in a more comprehensive depiction of tissue morphology [Fig. 4(c)]. Similar results were observed when the Mach–Zehnder interferometer was used for imaging. As clearly shown in Fig. 5(b), the hypointense artifacts were diminished when the pseudo-depolarizer was placed in the setup [Fig. 5(c)], leading to a more accurate picture of the tissue morphology and, consequently, improved image quality.

**Fig. 6:**
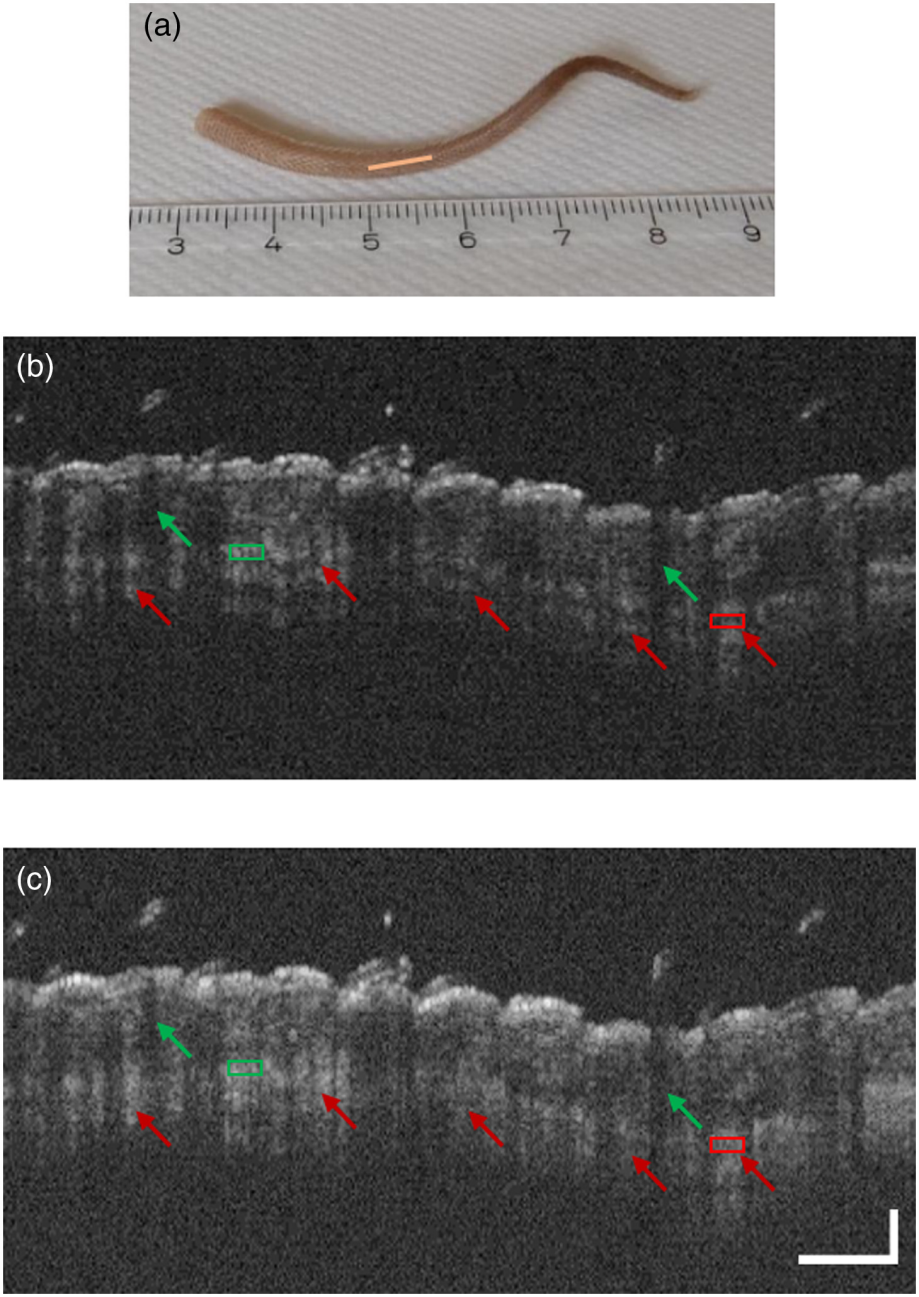
OCT imaging of a mouse tail specimen with polarization-insensitive OCT implemented in a fiber-based Michelson interferometer. (a) Scanned region of the mouse tail (orange line). Tissue imaging (b) without and (c) with the depolarization stage in the reference arm. The typical stripe pattern artifacts (red arrows) are drastically reduced. The vertical black lines (green arrows) are shadows originating from the hairs on the tail surface. Green and red boxes in panels (b) and (c) indicate the bright and dark ROIs, respectively, which were used for the SNR improvement quantification in the striped and hypointense tissue areas. The scale bars in panel (c) apply for both tomograms. The horizontal bar represents 750  μm, and the vertical bar represents 350  μm.

**Fig. 7:**
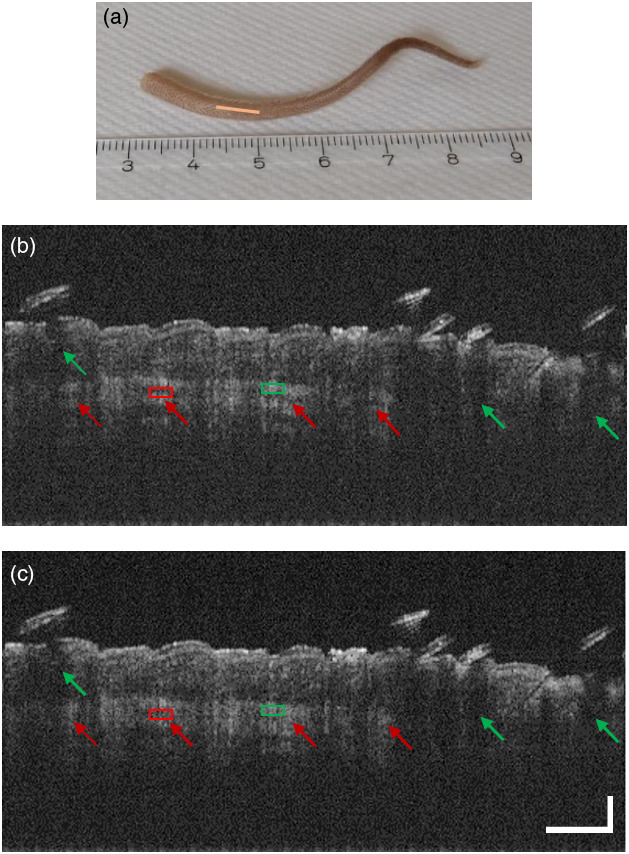
OCT imaging of the mouse tail specimen with polarization-insensitive OCT implemented in a fiber-based Mach–Zehnder interferometer. (a) Scanned region of the mouse tail. The tissue imaging (b) without and (c) with the optimal depolarizer configuration. After the DP implementation, the striped pattern artifacts are diminished, and the tissue structure appears clearer. The vertical black lines (green arrows) are shadows originating from the hairs on the tail surface. Bright ROIs in green and dark ROIs in red boxes were used for the SNR improvement quantification in striped and hypointense tissue areas, respectively. The scale bars in panel (c) apply for both figures. The horizontal bar represents 750  μm, and the vertical bar represents 350  μm.

Additional images were acquired from an *ex vivo* mouse tail specimen at locations shown in Figs. 6(a) and 7(a). As shown in Fig. 6(b), the typical stripe pattern artifacts caused by tissue birefringence were drastically reduced after the pseudo-depolarizer implementation in the reference arm of the Michelson interferometer [Fig. 6(c)]. Also, for the Mach–Zehnder setup, a similar effect on the suppression of these artifacts was observed, as shown in Figs. 7(b) and 7(c). By selecting regions of interest (ROIs) in the striped and hypointense tissue areas [Figs. 6(b) and 6(c) and Figs. 7(b) and 7(c)], the average SNR improvement was computed for the cases with and without the selected pseudo-depolarizer configuration as $10\;\log(I_{\max}/I_{\min})$, where $I_{\max}$ and $I_{\min}$ refer to the mean pixel intensity of the green and red boxes in Figs. 6 and 7 indicating the bright and dark ROIs of the stripe pattern, respectively. Specifically, a signal improvement of 3.2 and 3.1 dB was measured for the image data obtained with the Michelson and Mach–Zehnder setups, respectively.

## 4 Discussion

Our research investigated the efficacy of two liquid crystal depolarizers for mitigating artifacts in OCT imaging of scleral tissue. Birefringent tissues can alter the polarization state of light and cause cross-polarization–related OCT signal fading. For instance, previous OCT research focused on OCT imaging of the sclera revealed birefringence-related artifacts that resembled scleral vessels, distorting the imaging information.^27^^–^^29^ Recently, we demonstrated a PinS-OCT method providing polarization-insensitive reflectivity contrast in both free-space and fiber-optic OCT setups.^37^ Although the implementation was straightforward in bulk optics layouts, the modification of fiber-optic source arms required a free-space section and therefore led to inherent power loss in the source arm. Here, we presented an alternative approach using a depolarization module in the reference arm. Compared with our recently published method, this approach allows for more effective housekeeping of the available light power and also provides a much more accessible route to implement PinS-OCT in existing OCT setups, by simply adding one medium-cost optical element in the reference arm.

Two commercially available depolarizers, DP1 and DP2, were tested in different configurations, and based on systematic measurements using a QWP and a mirror as a variable sample, the most effective configurations were identified for two frequently used OCT layouts, namely, Michelson and Mach–Zehnder interferometers. The obtained graphs (Fig. 2) clearly illustrate the impact of the different pseudo-depolarizer configurations on the signal fluctuation and allow the identification of those configurations that result in the best polarization-related artifact suppression. The DOP values indicated in Fig. 3 can be used as complementary information by offering a broader overview of the overall depolarization effect caused by each configuration but do not always show a strictly monotonic correlation with artifact suppression. With the depolarization module engaged in the reference arm, the detected cross-polarized signal intensity was subject to significant improvement and thus reduced sample polarization-dependent fluctuations were observed. In particular, a substantial recovery of the relative signal fluctuations was observed for both the Michelson and the Mach–Zehnder setups. A notable artifact reduction after the depolarizer addition was also demonstrated in an imaging experiment in the scleral tissue of a post-mortem alpine marmot eye and mouse tail tissue. The hypointense artifacts were drastically reduced, whereas the tissue morphology appeared clearer with a more complete depiction of the tissue structure. Compared with our previous research^37^ where the depolarizer DP1 was placed in the source arm of a Michelson interferometer, we observed an almost similar improvement in the artifact reduction and signal intensity after the depolarizer addition, which highlights the stability of the approach in different OCT configurations. Although the artifact suppression and signal improvement using the depolarizer implementation in the reference arm was effectively achieved, a comparison of the results shown here reveals a slightly lower performance compared with our previous work.^37^ By comparing the data in Fig. 2 of the current work and Fig. S2 from our previous method, the relative signal fluctuations decreased from 5.7 to 1.8 dB for the reference arm implementation, whereas in the previous work, the fluctuations ranged from 4.7 to 1.3 dB. This difference could be partially attributed to the different pseudo-depolarizer configurations as well as to the partly different optical layouts. Moreover, for the configurations presented here, the sensitivity of the Mach–Zehnder interferometer decreased slightly from 93.3 to 92.7 dB after the DP addition in the reference arm, whereas for the Michelson interferometer, the sensitivity showed a stronger decrease from 95.1 to 93.3 dB for the implementation of the two depolarizers into the system. The introduction of the pseudo-depolarizers adds to the attenuation of the reference beam. Specifically, for the Mach–Zehnder interferometer, the reference beam was attenuated by 5%, whereas after passing through both depolarizers in the Michelson interferometer, the attenuation amounted to 12%. The observed sensitivity decrease may be attributed to reference power decrease as well as to the randomization of the reference polarization states after the implementation of the respective pseudo-depolarizer configuration in the reference arm of the OCT setups. However, particularly in fiber-based OCT systems, the reference arm approach offers additional advantages compared with the source arm approach. In fiber-optic interferometer layouts, the reference arm approach eliminates the need for a free-space section in the source arm to implement the depolarizer configuration. This not only simplifies the implementation but also reduces the overall power loss in the system as the free-space section required for the depolarizer implementation in a fiber-based interferometer would require coupling back into a single-mode fiber and would therefore result in an ∼50% loss of input power. Quantitative measurements in the striped and hypointense tissue areas support the positive effect of the proposed approach in the reduction of cross-polarization artifacts revealing a signal improvement of 3.2 and 3.1 dB after the pseudo-depolarizer implementation in the Michelson and Mach–Zehnder interferometers, respectively.

Alternative approaches based on polarization-diverse detection and PS-OCT have also targeted the elimination of image artifacts originating from birefringence and compared polarization-dependent with polarization-independent OCT images of scleral and lamina cribrosa tissue.^41^^,^^42^ Polarization-independent OCT methods can contribute to reduced cross-polarized artifact OCT imaging in tissues where their inherent birefringence can drastically alter the polarization state of incident light. Especially in tissues such as the muscles,^20^ tendons,^22^ skin,^21^ and white matter,^26^ PS-OCT has already been used to evaluate their birefringent properties arising from the collagen fiber orientation or nerve fiber microstructure. An approach based on mitigating the polarization-induced artifacts as the one presented in this paper can provide a more accurate tissue depiction when additional PS-OCT contrast such as retardation imaging is not needed. We further are positive that this method may also find applications in endoscopic OCT systems used for imaging the gastrointestinal tract or for intravascular OCT imaging procedures where birefringence artifacts are unavoidably introduced by probe twisting or bending during tissue imaging.^32^ Also, variations in temperature, external pressure, or vibrations may impact the optical properties of optical fibers and lead to alterations in their birefringence.^43^

Our study suggests several avenues for further research. Exploring the depolarization technique on other eye structures beyond the sclera or in non-ophthalmic applications, in particular, in other birefringent tissues such as the aforementioned, holds the potential for a wider clinical influence and advancements in biomedical imaging. Also, the investigation of alternative depolarizer configurations such as calcite or magnesium fluoride depolarizers and the implementation of more sophisticated depolarizer designs tailored to perfectly match the wavelength characteristics of the OCT systems used may be worthwhile and offer optimized artifact reduction for PinS-OCT. In general, the practical implementation of this method makes it suitable and easily applicable in fiber-based OCT systems which are mostly used in research. As prominent applications, OCT imaging systems for ophthalmic and endoscopic applications may be upgraded to even more advanced diagnostic tools in the clinical routine by benefitting from the easy implementation and effectiveness of our PinS-OCT approach. We believe that this simple yet inexpensive PinS-OCT approach is a promising technique that can provide improved OCT image quality with a more complete tissue representation and reduced artifacts for a wide range of biomedical imaging applications.

## 5 Conclusion

We presented a simple approach for polarization-insensitive OCT and demonstrated its efficiency in reducing OCT image artifacts caused by tissue birefringence. By implementing a depolarization module in the reference arm of the Michelson and Mach–Zehnder interferometers, these artifacts were drastically reduced, leading to an enhanced OCT image quality and a more complete depiction of scleral tissue. Our research highlights the effectiveness of a simple and cost-effective technique that can be easily integrated into existing OCT technology in various biomedical fields and may significantly improve diagnostic information.

## Data Availability

Code and data underlying the results presented in this paper are not publicly available at this time but may be obtained from the authors upon reasonable request.
